# Perinatal Complications Associated With Teenage Pregnancy at the IMSS-Bienestar Specialties Hospital “Dr. Carlos Canseco”, Tampico, Mexico: A Case-Control Study

**DOI:** 10.7759/cureus.77198

**Published:** 2025-01-09

**Authors:** Marco Antonio Robles Mejía, Mario Alberto Maldonado Ramírez, José M Torres-Benítez, Josué Robles Vázquez

**Affiliations:** 1 Obstetrics and Gynecology, IMSS-Bienestar Tampico Specialty Hospital “Dr. Carlos Canseco”, Tampico, MEX; 2 Pediatric Dentistry, Autonomous University of Tamaulipas, Tampico, MEX; 3 Public Health, IMSS-Bienestar Tampico Specialty Hospital “Dr. Carlos Canseco”, Tampico, MEX; 4 Medicine, Autonomous University of Tamaulipas, Tampico, MEX

**Keywords:** adolescents, adults, complications, perinatal, pregnancy

## Abstract

Background: Adolescent pregnancy has been increasing recently, becoming a focus of alarm for health since it presents greater risks and complications for both the mother and fetus, putting the lives of both at risk.

Objective: To identify the maternal-obstetric and perinatal complications that occur in pregnancy in women under 19 years of age compared to those in adults, and to determine whether age is a risk factor for greater complications.

Methods: This observational, analytical, and retrospective study included 239 patients with resolution of pregnancy - 92 adolescents and 147 adults - who met the selection criteria for the case-control study.

Results: A total of 37% (34) of the adolescents had inadequate prenatal control, compared to 34% (49) of the adults. Of the adolescents, 10.9% (10) had miscarriages, 8.7% (8) had urinary tract infections (UTIs), and 2.2% (2) had tears at delivery, which were the most frequent complications. A total of 39.5% (58) of the adults had cesarean sections; the variety of fetal position and amount of amniotic fluid only occurred in 2.0% (3) and 8.8% (13) of their products, respectively.

Conclusion: The most frequent maternal complications are miscarriages, UTIs, and hemorrhages, all of which present in a higher percentage of adolescent pregnancies. Maternal complications were more frequent in adolescents; however, the products of adult mothers had more complications.

## Introduction

Pregnancy in adolescents has been increasing recently and has been a focus of health alarm, according to Bendezú et al. (2015) and Ortiz et al. (2018) since there are greater risks and complications than in adult women, with anemia, urinary tract infections, and premature rupture of membranes prevailing, as well as cesarean sections and injuries to the birth canal such as genital tears [1,2]. In addition, Sandoval et al. (2007), Ortega-Castro (2017), and Velasteguí-Egüez et al. (2018) remark that adolescence is an important risk factor for maternal complications, including hypertensive disorders, chorioamnionitis, and preterm births. Therefore, it is crucial to identify pregnant adolescents, monitor them, and have greater prenatal control to avoid unwanted outcomes [3-6]. Most of the complications presented have had more significance for the 12- to 15-year-old group; however, it is important to note that they can occur at most early ages, considered as adolescent pregnancy, which implies adverse problems for both the mother and fetus, impacting the health of both [4]. Also, Villalobos-Hernández et al. (2015) refer to the main perinatal complication that has been seen in adolescent pregnancies as abortion, followed by cephalopelvic disproportion and fetal distress. It has been observed that there are a series of causal factors involved that negatively favor these complications, mainly young age, socioeconomic status, and a low level of education [7].

Although pregnancy during adolescence is more worrying than at older ages, according to Cortez-Anyosa and Díaz-Tinoco (2020), the risk of complications occurs in both adolescents and older adults [4,8-11]. Maternal mental, emotional, and biological immaturity together with a lack of awareness or interest in prenatal care may increase the risk of complications during delivery. This highlights the importance of prenatal monitoring to help prevent deficiencies that may lead to complications [12-14]. Most adolescents do not have adequate prenatal care; only 18% had care throughout their pregnancy. That is why the best approach to the exposed problem is the prevention of pregnancy in adolescents, especially those under 15 years of age, in whom perinatal maternal indicators are more unfavorable [15].

Perinatal maternal complications are a crucial factor in younger adolescents since they are related to maternal death four times more frequently in early adolescents than those over 15 years of age, in whom there is no greater risk. In late adolescence, various psychological and social factors are seemingly much more relevant and have a greater impact [5]. The age of teenage mothers is, on average, 17 years old. The probability of maternal mortality is twice as high in children under 15 years of age as in women 20 years of age or older. Likewise, children born to women under 20 years of age are at greater risk of dying before their first year of life. Therefore, the younger the mother is, the probability of maternal and neonatal mortality increases [6]. The medical risks to adolescent mothers also cause an increase in infant mortality, which can double or even triple due to the physiological and anatomical immaturity of the mother. Adequate care during pregnancy can minimize unfavorable outcomes, although this is not the case in all cases [8-11]. On the other hand, according to Macías-Villa et al. (2018), newborns of older adult mothers have more complications after birth [16].

The objective of this study is to identify the maternal-obstetric and perinatal complications that occur in pregnancy in women under 19 years of age compared to those that occur in adulthood to determine if age is a risk factor for greater complications in the region of Mexico.

## Materials and methods

The present work is an observational, analytical, and retrospective study of cases and controls, acknowledged by the Research Ethics Committee (176/2024/CEI-HGT), where the maternal-perinatal complications that occurred during pregnancy and newborn of adolescent and adult women were compared to determine if age is a risk factor for the development of greater complications.

Two study groups were formed: pregnant adolescent women aged less than or equal to 19 and pregnant women during adulthood over 20 years old.

To carry out this study, the clinical records of the women who had a pregnancy resolution in the Tocosurgery Department Service of the General Hospital of Tampico “Dr. Carlos Canseco” during June and July 2022 were used.

The study variables that were classified as dependent were age, marital status, education, alcoholism, smoking, drug addiction, tattoos, height, weight, BMI, obesity, and maternal comorbidities as well as prenatal controls, the number of ultrasounds performed, single pregnancies, births, abortions, and cesarean sections, as well as the birth route, the weeks of gestation and whether the neonatal was preterm, the Apgar score, whether it was a single pregnancy as well as its weight and height.

A non-probabilistic sampling of consecutive cases was considered for convenience; therefore, the sample size was 239 women - 92 adolescents and 147 adults - who met the selection criteria for the case-control study. The inclusion criteria were age equal to or less than 19 years old, and adults aged 20 years and older who were treated with the resolution of the pregnancy in June and July and hospitalized in the Tocosurgery Service. The exclusion criteria were women admitted to the Tocosurgery Service who had a diagnosis other than labor. And the elimination criteria were women who had an incomplete file, a voluntary discharge, or a transfer to another hospital service.

The information collected, laboratory data, and clinical records of the women were managed with confidentiality, following the principles of the Helsinki Declaration and the general health law. Once the information was obtained, a database was designed in Excel Office (Microsoft® Corp., Redmond, WA, USA) for statistical analysis using descriptive statistics, the distribution of frequencies and percentages for nominal variables, and subsequently the statistical differences with the Student's t-test and chi-square test. The Statistical Package for the Social Sciences (IBM SPSS Statistics for Windows, IBM Corp., Version 20.0, Armonk, NY) and Epi Info 3.5.1 (Centers for Disease Control and Prevention (CDC), Atlanta, USA) were used.

## Results

There were 354 cases of pregnancy resolution recorded during the study period; however, 45 files were not found in the archives department, so only 309 files were reviewed. A total of 60 records were excluded, 16 of them for not meeting inclusion criteria, 18 for having a hospitalization with a diagnosis other than labor, and 26 for having incomplete records. In the end, a sample of 249 files was obtained, which constituted the case and control groups under study.

The average age of the study population, both cases and controls, was 23 years (range 13 to 40 years), with the group of cases being adolescents with an average age of 17.3±1.3 years with a range of 13-19 years, and the average age of the control group was 27.3±5.0 years, with a range of 20-40 years.

Education of pregnant adolescents compared to pregnant adults

A higher percentage of pregnant women with high school education is observed, with 53.3% (48) of adolescents and 45.6% (67) of adults with high school education, with adolescents being the ones who most frequently become pregnant during this stage. In both groups, there is an almost equal frequency of pregnant women in high school, with 34.7% (49) of adults and 32.6% (30) of adolescents with this education. Primary education prevails in adolescents with a frequency of 14.1% (13), unlike adults with 8.2% (12), but with the difference that in this age group, there is a higher percentage with a sophomore education than primary education, with 11.6% (17) of adults over 20 years old who have a higher education (p<0.05).

Prenatal control in pregnant adolescents in comparison with adults

When compared to both groups, there is a higher percentage of pregnant women who follow proper prenatal care: 66% (98) of adult women and 63% (58) of adolescents, but adult women have a greater level of prenatal control, with an odds ratio of 1.14 and a 95% confidence interval (CI) of 0.66-1.96 (p>0.05).

It has been observed that labor is the most common way to births in both groups (Table 1), but more frequently in adolescents, with 68.5% (63) of births being done via labor; the difference in the birth route in newborns was 16.8% between both groups. Unlike adult women, in whom a percentage of 39.5% (58) of births by cesarean section is observed, this method of birth is higher in adults than in adolescents. Abortions were more frequent in adolescent pregnancies, with 10.9% (10), unlike the 8.8% (13) of abortions in adults (p<0.01).

**Table 1:** Route of birth by groups

Resolution	Teenagers	%	Adults	%	p-value
Abortion	10	10.9	13	8.8	0.4958
Cesarean Section	19	20.7	58	39.5	0.0025
Vaginal Birth	63	68.5	76	51.7	0.0105
Total	92	100.0	147	100.0	

Table 2 displays the most frequent maternal complications that occurred in adolescents and adults, and it is evident that in both groups, more than half did not experience these complications: adults 53.7% (79), adolescents 52.2% (48), with a minimum difference of 1.5%. Abortions were the complication that occurred mostly in adolescents at 10.9% (10), as were urinary tract infections at 14.1% (13), with adolescents being more frequent compared to 10.9% (16) of adults. Hypertensive diseases were the third most frequent complication, occurring mostly in 11.6% (17) of adults, with a difference of 4% (10) more than in adolescents.

**Table 2:** Maternal complications present in pregnant adolescents and adults

Maternal Complications	Teenagers	%	Adults	%	p-value
Abortion	10	10.9	13	8.8	0.6053
Urinary Infection	13	14.1	16	10.9	0.4546
Perineal Tear	2	2.2	0	0.0	0.0726
Hypertensive Disorders	7	7.6	17	11.6	0.3221
Uterine Hypotonia	1	1.1	5	3.4	0.2658
Obstetric Hemorrhage	4	4.3	6	4.1	0.92.3
Premature Rupture of Membranes	7	7.6	7	7.5	0.3618
None	48	52.2	79	53.7	0.8132
Total	92	100.0	147	100.0	

Table 3 displays the various complications that occurred during the birth of newborns of adolescent and adult mothers, and we noticed that in both groups, the majority were born without any complications: adolescents 70.7% (65) and adults 65.3% (96); however, the newborns of adolescents were the ones who had the fewest complications compared to adults, with a difference of 5.4%. On the contrary, there were more premature newborns in adults (9.5%, n=14) than in adolescents (8.7%, n=8), but by a minimal difference of 0.8%. Acute fetal distress was the complication that occurred more in the newborns of young mothers (3.3%, n=3) than in those of adult mothers (2.0%, n=3). Low weight and stillbirths occurred in the same proportion only in adolescent mothers (2.2%, n=2), unlike the variety of fetal positions that only occurred in adults (2.0%, n=3). Another serious complication that draws the most attention is the variety of amounts of amniotic fluid that only manifest in the products of adult mothers (8.8%, n=13), of which oligohydramnios, polyhydramnios, and anhydramnios stand out.

**Table 3:** Complications in newborns by group

Neonatal Complications	Teenagers	%	Adults	%	p-value
Abortion	10	10.9	13	8.8	0.6053
Low Fetal Reserve	1	1.1	1	0.7	0.7370
Low Birth Weight	2	2.2	0	0.0	0.726
Neck Circular	1	1.1	4	2.7	03904
Fetal Mortality	2	2.2	0	0.0	0.0726
Premature	8	8.7	14	9.5	0.8294
Acute Fetal Distress	3	3.3	3	2.0	0.5574
Polyhydramnios	0	0.0	13	8.8	0.0034
Position Variety	0	0.0	3	2.0	0.1679
None	65	70.7	96	65.3	0.3911
Total	92	100.0	147	100.0	

## Discussion

Teenage pregnancy is defined as one that occurs before the age of 20. In this study, we consider cases of pregnancies that occurred in women aged 19 years or younger. Álvarez-Huante et al., in their study, found that the average age of pregnant adolescents was 17 years old [11], which agrees with the results of this work.

The appearance of teenage pregnancies influences the academic level since these young women are usually forced to drop out of their studies, thus having a poor educational level. Villalobos-Hernández et al. mentioned that there is an association between the occurrence of pregnancy and the probability of dropping out of school [12], which can be observed in our study since most of the population studied has high school as the highest level of study: adolescents (53.3%, n=49) and adults (45.6%, n=67), prevailing more in adolescents due to pregnancy during stages of basic educational levels.

Prenatal control is aimed at carrying out systematic or periodic actions and procedures to prevent, diagnose, and early treat obstetric risk factors that may cause maternal and perinatal morbidity. Leiva-Parra E et al. pointed out that a higher percentage of adolescents have poor prenatal care [13], coinciding with the results of our study, in which we can observe that a higher percentage of young people (37%, n=34) compared to adults (34%, n=49) do not attend the minimum of five consultations established by the World Health Organization to consider optimal prenatal care.

Jiménez-Cabañas et al. observed in their study that in both adolescents and adults, the route of birth was by vaginal delivery. However, cesarean sections were more frequent in women over 20 years of age [14], which we agree with in this study since, based on our results, it was found that childbirth was more prevalent with 68.5% (63) of adolescents and 51.7% (76) of adults in the general study population, but that, unlike adolescents, a higher percentage of adults had cesarean sections, which in turn is related to maternal complications. Only adults reported variations in fetal position (2.0%, n=2) and the amount of amniotic fluid (8.8%, n=13), which contributed to cesarean section being preferred over vaginal delivery for resolving the pregnancy. In contrast, among adolescents, the frequency of perineal tears was 2.2% (n=2), with this group being the only one to experience this complication, likely due to underdevelopment of the maternal pelvis.

García-Salgado et al. reported that the main maternal complications among adolescents were urinary tract infections (UTIs) and abortion [15]. Similarly, in the present study, UTIs were observed in 8.7% of cases and abortions in 10.9%, aligning with their findings and confirming that these are the most common complications experienced by young mothers.

There is evidence that the older the woman, the greater the maternal complications. Macías-Villa et al. concluded that the complications that occurred most in adult mothers were hypertensive diseases such as preeclampsia, eclampsia, and gestational hypertension, as well as preterm births [16]. This concurs with the results obtained in this study.

Although the sample size is limited, the study provides us with insight into the factors that affect maternal and perinatal complications in adolescent mothers.

## Conclusions

The poor education in both groups is evident, with high school being the highest level of academic level they have, which is more prevalent in adolescent girls due to their young age. Adolescents have poor prenatal care, unlike adults, which could influence the complications that occur in both the mother and the child. The most prevalent route of birth in both groups is vaginal birth; however, compared to adolescents, in adults, there is a higher percentage of births by cesarean section. The most frequent maternal complications are abortions, UTIs, and hemorrhages, all of which occur at a higher percentage in adolescent pregnancies.

The majority of the population, including both cases and controls, had no complications in their newborns; however, comparing both groups, the newborns of adult mothers had greater complications, the most frequent being oligohydramnios, prematurity, and the variety of positions of the fetus. It was observed that maternal complications were more frequent in adolescents; however, the newborns of adult mothers had more complications.
